# A Pilot Randomized Controlled Trial to Evaluate Isosorbide Mononitrate (IMN) Efficiency for Cervical Ripening Prior to Labor Induction in Iranian Pregnant Women

**DOI:** 10.22037/ijpr.2017.2040

**Published:** 2019

**Authors:** Shokohosadat Abotorabi, Masoome Mohammadi, Samira Bagherivand, Sonia Oveisi

**Affiliations:** a *Department of Obstetrics and Gynecology, School of Medicine, Qazvin University of Medical Sciences, Qazvin, Iran. *; b *Department of Metabolic Diseases Research Center, School of Medicine, Qazvin University of Medical Sciences, Qazvin, Iran.*

**Keywords:** Isosorbide mononitrate, Bishop score, Labor, Term pregnant, Cervical ripening

## Abstract

Our aim was to examine the effect and cost-effectiveness of isosorbide mononitrate (IMN) for outpatient (at home) cervical ripening in Iranian women with term pregnancy. Term pregnant women were randomly selected to receive either 15 mg vaginally administered IMN or placebo. Patients were advised to take the second dose at home 24 h later and return to the hospital for admission after the next 24 h if the onset of spontaneous contraction does not occur. Cervical status, maternal blood pressure, maternal pulse rate, fetal heart rate and various side effects were examined. IMN induced a significant increase in cervical Bishop Score; headache, nausea, and dizziness were seen in 8 of 28 participants of IMN group; although, all had normal vital signs and normal FHR, they had to be hospitalized and receive medication to relieve the unpleasant symptoms. Vaginal administration of 15 mg of IMN induced cervical ripening in term pregnant women. However, the administration of IMN for cervical ripening is not recommended as it produced headache in significant number of Iranian pregnant women.

## Introduction

Labor induction in term pregnancy in the presence of an unfavorable cervix is often prolonged and this leads to longer hospitalization before delivery and unsuccessful induction consequences in an operative delivery (1). Approximately, in 20% of pregnant women undertaking induction of labor, the mean time taken from induction to delivery is between 15 to 20 h, of which up to 12 h are spent in the cervical ripening phase before the beginning of labor (2). The achievement of labor induction is associated with cervical ripening and various prostaglandin regimens are usually employed to ripen the cervix. Prostaglandin may cause adverse fetal and maternal effects. It is estimated that about 5% of women experience uterine hypertonus following the administration of prostaglandin (3). An ideal ripening agent should make the cervix soft, compliant and moderately dilated without stimulating uterine contractions and with no clinically important side effect in the mother or her baby (4). IMN is a nitric oxide (NO) donor and vasodilator mainly used for patients with angina pectoris (5).

Nitric oxide has a multifunctional role in inflammation with various pro-inflammatory effects including an increase in vascular permeability, cytotoxicity and tissue damage, changes in glycosaminoglycan synthesis, and apoptosis. Apoptosis is recognized to play an important role in cervical ripening (6). The nitric oxide system relaxes the myometrium and nitric oxide donors have been proposed as tocolytic agents in the management of preterm labor and effectively been used in treating intrapartum fetal distress caused by uterine hypertonus (7).

Therefore it is important to study the clinical effect of local treatment with nitric oxide donors on the uterine cervix and assess the probable adverse maternal and fetal effects of this medical treatment at term (8).

Several trials have established the efficacy and safety of vaginally administered IMN but 3 randomized clinical trials using IMN for cervical ripening in term pregnancy produced varying degrees of success and outcome in Iran (9-11).

Due to the discrepancy between the research in Iran and recent recommendations to promote and facilitate NVD, the need for further research in this field is felt. The present study was carried out to evaluate the safety, efficacy, and side effect of self-administered IMN treatment for cervical ripening in Iranian outpatient women with term pregnancies.

## Experimental

This was a pilot randomized double-blind, placebo-controlled study (IRCT = 201301134850N2) conducted at Kosar teaching hospital, Qazvin (Iran) from October 2012 to February 2014. The study was approved by the Human Ethics Committee of Qazvin University of Medical Sciences and all pregnant women included in the study provided written informed consent to enter the study.

*Participants*

Inclusion criteria were maternal age of 18 years or older, unfavorable cervix (Bishop score 6 or less), uncomplicated singleton pregnancy with vertex presentation, reactive non-stress test, gestational age between 38 and 42 weeks and absence of labor, normal amniotic fluid index measuring ≥5 cm, intact fetal membranes and absence of contraction frequency of three or greater in 10 min as well as absence significant systemic maternal disease other than preeclampsia or diabetes.

Exclusion criteria included consisted of fetal malpresentation, cesarean repeat or uterine surgery and presence of a placenta previa or low-lying placenta, contraindications to receive nitric oxide and prostaglandins (allergy, history of severe asthma, hypotension, palpitation), regular uterine contractions, cardio respiratory disease, history of headache, intolerance to isosorbide mononitrate, and serious disease defined as daily use of medication (3, 5 and 12).

**Figure 1:**
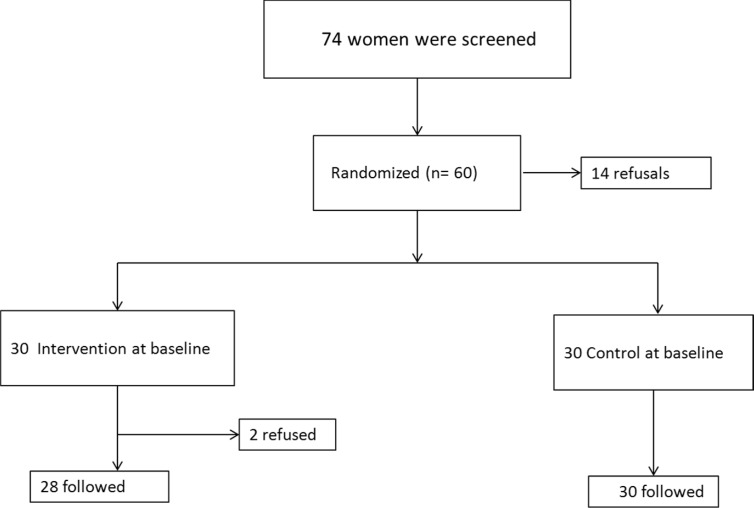
Flow chart of the study participants

The intervention group received IMN 15 mg (Dexel Pharma, UK) and the control group was given placebo tablets of similar design as isosorbide mononitrate. Figure 1 shows the trial profile and the proportion of participants who completed follow-up. Two patients were excluded from the study because they gave birth in a different hospital. Among the remaining 58 women, 28 were in the IMN group and 30 in the control group.

*Procedure*

Randomization was performed through random-number tables in which the sorting blocks were in sealed opaque envelopes. Both the IMN and the placebo tablets were administered into the posterior vaginal fornix by a research nurse. Neither the participating women nor the research nurse were aware of the agent administered.

First of all, it should be noted that before the beginning of the study, we used IMN 30 mg tablets in 6 persons as pilot, but within the 24 h following the administration of medications, the patients complained of painful headaches (7). Therefore, the authors were forced to decrease the dose of IMN to 15 mg. Later, based on the blocks and while the patients were at the gynecology outpatient clinic, IMN 15 mg and placebo were placed inside the vagina of intervention and control groups, respectively.

The patients were advised to take the second dose at home after 24 h and return to the hospital for admission after the next 24 h if the onsets of spontaneous contraction do not occur.

*Measures*

Assessment of the cervix, based on other references, included consistency, length, dilatation, position, and station of the fetal presenting part as first described by Bishop Score (8).

In addition, maternal pulse rate and blood pressure, fever, headache, and palpitation were recorded. If inclusion criteria were fulfilled, a signed informed consent form was obtained from each participant before recruitment. If labor contractions did not start, the induction of labor would begin. Labor, birth length, the causes of cesarean, weight, sex, and the Apgar score at 1 and 5 min for all newborns were recorded.

*Statistical analysis*

We first compared the demographic characteristics of the two study groups using chi-squared and *t*-tests. There was no significant difference in the demographic variables between the two groups (Table 1). The intervention effects were analyzed by repeated measures ANOVA with dependent measures, described earlier in the Measures section.

## Results and Discussion

Of 70 eligible women approached for the study, 60 provided signed informed consent form and 10 declined enrolments. Maternal demographic and obstetric characteristics were similar between the two groups with no difference in gestational age (Table 1).

The indication for induction of labor (as defined by the obstetrician caring for the woman) was postdating pregnancy in all women. There was no difference in the indication for induction of labor between the two study groups. There was no tachysystole or hyperstimulation in our study groups. Headache, nausea, and dizziness were seen in 8 of 28 participants of the IMN group, although with normal vital signs and normal FHR, they were hospitalized and needed medication to relieve the symptoms (Tables 2 and 3).

Neonatal outcomes including birth weight, birth weight greater than 4,000 g, Apgar scores less than 7 at 1 and 5 min, meconium at the time of ruptured membranes, and neonatal intensive care unit admission were similar between the two groups. However, no baby of either group was admitted to NICU.

**Figure 2:**
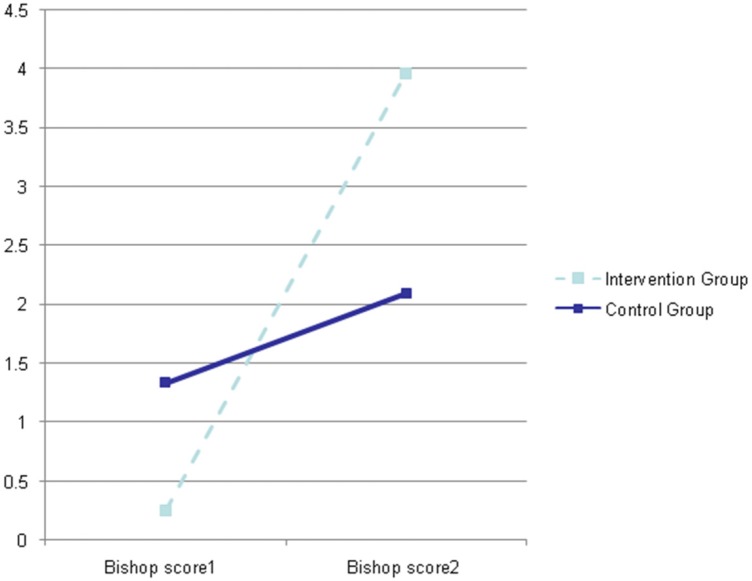
Comparison of Bishop Scores in two stages between intervention and control group by repeated measures ANOVA

As shown in Figure 2, the Bishop Score in the IMN group was 0.2 ± 0.5 at the first examination and 3.96 ± 1.4 at the sec examination whereas in the control group the Bishop scores were 1.33 ± 0.8 and 2.1 ± 1.7 at the first and second examinations, respectively (*p *= 0.001; F = 53.76).

Furthermore, we were interested in calculating the strength of treatment effect with isosorbide (effect size) in our intervention and control groups which was estimated using Cohen’s guideline, whereby a value of 0.2 denotes a small, 0.5 a medium, and 0.8 a large effect size (13).

The results obtained in our study showed that the effect size of the present study was 2.77, indicating a large effect size in the IMN group with Bishop score equal or higher than 6 in 5 cases (17.9%) of IMN group and 2 cases (6.7%) of control group, however, no significant difference (*p* = 0.19) was observed between the two groups while 3 cases (11%) lacked the onset of spontaneous contraction 48 h following the administration of medication.

**Table 1:** Characteristics of study participants by *t*-test measure

**Characteristic**	**Isosorbide mononitrate Mean ± SD (n = 28)**	**Placebo Mean ± SD (n = 30)**	***P***
Maternal age (year)	22.04 ± 3.82	22.8 ± 4.23	0.49
BMI	28.71 ± 4.61	30.29 ± 4.21	0.17
Gestational age (week)	40.14 ± 0.32	40.09 ± 0.45	0.5
Induction time	6.63 ± 5.38	6.09 ± 5.23	0.7

**Table 2.** Between-groups comparison of labor and parity by chi-square measure

**Outcome**	**isosorbide mononitrate (n = 28)**	**Placebo (n = 30)**	***P***
Delivery Mode, VD	18 (64.3%)	16 (53.3%)	0.04
Nulliparity N (%)	21 (50%)	21 (50%)	

**Table 3:** Compare of maternal side effects between two groups by chi-square measure

**Variable**	**Group**	**Yes**	**No**	**Total**	***P*** **-value**	**Χ** **2**
	case	8 (28.6%)	20 (71.4%)	28 (100%)		
Headache						
	Control	0 (0%)	30 (100.0%)	30 (100%)		
					0.002	9.94
	case	0	28 (100.0%)	28 (100.0%)		
Palpitation						
	Control	0	30 (100.0%)	30 (100.0%)		
	case	1 (3.6%)	27 (96.4%)	28 (100.0%)		
Fever					0.296	1.09
	Control	0	30 (100.0%)	30 (100.0%)		

**Table 4:** Comparison of labor complications between-groups by chi-square measure

**variables**		**Failure to progress**		**Fetal distress**		**Meconium**		
		**yes**	**no**	**yes**	**no**	**yes**	** no **	*P*-value, Χ2
Drug	yes	3 (10.7%)	25 (89.3%)	4 (14.29%)	24 (85.71%)	2 (7.1%)	26 (92.9%)	0.734, 2.01
	no	6 (20%)	24 (80%)	4 (13.3%)	26 (86.7%)	4 (13.8%)	26	(86.2%)

Palpitation was not observed in any members of the IMN or control groups. The induction time in the IMN group was 6.63 ± 5.38 h and in control group 6.09 ± 5.24 h with no significant difference between the two groups (*p* = 0.785). 

 Normal vaginal delivery (NVD) occurred in 18 (64.3%) cases of the IMN group and 16 (53.3%) cases of the control group with no statistically significant difference between the two groups (*p* = 0.4). The indications for cesarean section in the IMN and control groups were fetal distress (4 versus 4 cases), failure to progress (3 versus 6 cases), and meconium (2 versus 4 cases), respectively (Table 4).

## Conclusion

This study was conducted according to the request made by pregnant women to decrease the period of hospital stay and also to consider the fact that many studies have shown that IMN can induce cervical ripening. This study examined the effects of IMN in term pregnant mother and fetus.

Economic evidence in the area of induction of women generally proposed that an unfavorable cervix prior to induction of labor significantly increases the rate of caesarean delivery. As caesarean delivery is the most costly type of delivery (14). 

This could be due to the reduced dose used in the present study based on the data found in our pilot review which was lower than the dose used in previous studies. However, it is noteworthy that although the repeat measure showed a significant difference in Bishop score between the two groups at the primary evaluation, nevertheless, the difference in the effect size was found to be obvious after the secondary evaluation even though the women treated with IMN had a higher mean Bishop score after 48 h compared to the women given placebo tablets at the appointment which indicated a significant difference in Bishop score between the two groups.

Results of another study demonstrate the important findings in outpatient cervical ripening using IMN in term pregnancies. Outpatient use of IMN 36 h before hospital admission for induction of labor meaningfully shortened the time from hospital admission to delivery. There was also a significant difference in Bishop scores between groups at the time of admission (15). 

The current study failed to evaluate the effects of this drug in creating hyperstimulation due to its lower dose used. However, we did not observe any adverse fetal heart after the administration of IMN and the fetal distress affecting the two study groups was shown to be similar. Also, there was no significant difference in the mode of delivery, Apgar score, and the need for neonatal intensive care was found between women treated with IMN and women who were given placebo tablets. Side effects of headache, tachycardia and a fall in maternal blood pressure are probably associated to circulate levels of isosorbide mononitrate. In other words, the ripening effects of vaginally administered nitric oxide donors are probably due to a local action of the drug, but side effects are due to systemic absorption (16).

Pregnant women are interested in self-administrating the drugs used for softening of cervix at home and before admission to hospital and this could benefit the mental health of mothers and reduce their hospitalization cost. However, isosurbide mononitrate despite the softening effect on cervix and its low dose cannot be recommended for pregnant women as it induces a complaining headache, leading to hospitalization of patients.

Considering the importance of assessing the effect of drugs on individuals in different countries for adjusting the most appropriate dose of the drug, isosurbide mononitrate, at least at the recommended dose used in other studies, cannot be prescribed as a drug for ripening of cervix in the pregnant women of Iranian population. 
